# How to Capitalize on the Retest Effect in Future Trials on Huntington’s Disease

**DOI:** 10.1371/journal.pone.0145842

**Published:** 2015-12-29

**Authors:** Catherine Schramm, Sandrine Katsahian, Katia Youssov, Jean-François Démonet, Pierre Krystkowiak, Frédéric Supiot, Christophe Verny, Laurent Cleret de Langavant, Anne-Catherine Bachoud-Lévi, European Huntington's Disease Initiative Study Group and the Multicentre Intracerebral Grafting in Huntington's Disease Group

**Affiliations:** 1 INSERM U955 E01, Neuropsychologie interventionnelle, Institut Mondor de Recherche Biomédicale, Créteil, France; 2 INSERM UMRS1138 E22, Science de l'information au service de la médecine personnalisée, Centre de Recherche des Cordeliers, Université Paris 5, Université Paris 6, Paris, France; 3 Université Paris Est, Faculté de Médecine, Créteil, France; 4 Ecole Normale Supérieure, Institut d’Etude de la Cognition, Paris, France; 5 Assistance Publique-Hôpitaux de Paris, Service d’informatique et statistiques, Hôpital Européen Georges Pompidou, Paris, France; 6 Assistance Publique-Hôpitaux de Paris, Centre National de Référence pour la Maladie de Huntington, Hôpital Henri Mondor, Créteil, France; 7 Leenaards Memory Centre, Clinical Neurosciences Department, CHUV Lausanne, Lausanne, Switzerland; 8 Centre Hospitalier Universitaire d'Amiens, Service de neurologie, Amiens, France; 9 EA 4559 - Laboratoire de Neurosciences Fonctionnelles et Pathologie (LNFP), Université de Picardie Jules Verne (UPJV), Amiens, France; 10 SFR CAP-Santé (FED 4231), Amiens, France; 11 Hôpital Erasme ULB, Service de Neurologie, Bruxelles, Belgium; 12 CHU d'Angers, Centre de Référence des Maladies Neurogénétiques, Service de Neurologie, Angers, France; Inserm U837, FRANCE

## Abstract

The retest effect—improvement of performance on second exposure to a task—may impede the detection of cognitive decline in clinical trials for neurodegenerative diseases. We assessed the impact of the retest effect in Huntington’s disease trials, and investigated its possible neutralization. We enrolled 54 patients in the Multicentric Intracerebral Grafting in Huntington’s Disease (MIG-HD) trial and 39 in the placebo arm of the Riluzole trial in Huntington’s Disease (RIL-HD). All were assessed with the Unified Huntington’s Disease Rating Scale (UHDRS) plus additional cognitive tasks at baseline (A_1_), shortly after baseline (A_2_) and one year later (A_3_). We used paired *t*-tests to analyze the retest effect between A_1_ and A_2_. For each task of the MIG-HD study, we used a stepwise algorithm to design models predictive of patient performance at A_3_, which we applied to the RIL-HD trial for external validation. We observed a retest effect in most cognitive tasks. A decline in performance at one year was detected in 3 of the 15 cognitive tasks with A_1_ as the baseline, and 9 of the 15 cognitive tasks with A_2_ as the baseline. We also included the retest effect in performance modeling and showed that it facilitated performance prediction one year later for 14 of the 15 cognitive tasks. The retest effect may mask cognitive decline in patients with neurodegenerative diseases. The dual baseline can improve clinical trial design, and better prediction should homogenize patient groups, resulting in smaller numbers of participants being required.

## Introduction

Huntington’s disease (HD) is an inherited neurodegenerative disorder involving motor, behavioral and cognitive impairments [1]. The cognitive disorders have a major impact on daily life, but most clinical trials focus on motor endpoints. This is because clinical trial endpoints must be able to capture both patient decline and treatment efficacy, and cognitive decline is much more difficult to capture within one year in patients at early disease stages [2] than motor decline. This difficulty of assessment results from the heterogeneity of cognitive changes (language, memory, etc.) and two opposing effects: the retest effect and patient decline due to disease progression. The retest effect is defined as an improvement in performance with repeated exposure to a task, with the greatest improvement occurring between the first two assessments [3–5]. This effect combines familiarity with the task and testing environment and the possible recall of responses [2]. The first assessment, during which everything is new to the patient, is always the most difficult.

The retest effect may have contributed to the failure of some neuroprotection trials, by adding noise to statistics comparing patients with different backgrounds at baseline, particularly in trials including small numbers of patients, such as those assessing biotherapy. One approach to neutralizing the retest effect is to carry out a second assessment (A_2_) shortly after the first (A_1_), and then discard the results obtained at A_1_ from the analysis, using performance at A_2_ as the baseline [2]. In addition, the retest effect (ΔA_2_-A_1_) can be used to improve the prediction of long-term patient performance. Indeed, in an observational longitudinal study in HD patients, the retest effect (ΔA_2_-A_1_ around 7 months) accounted for up to 36% of the variance of performance at A_3_ (ΔA_3_-A_2_ around 29 months) [6]. Likewise, in healthy elderly adults, performance at A_3_ (one year) is accurately predicted by the one week-interval retest effect (ΔA_2_-A_1_) [7].

However, the impact of the retest effect in clinical trials, which include additional variability (placebo effect, hope, anxiety about treatment and randomization), remains unknown. Two trials, the *Multicentric Intracerebral Grafting in Huntington’s Disease* (MIG-HD) [8] and *Riluzole in Huntington’s Disease* (RIL-HD) [9] trials, were designed with a short-term test-retest procedure. We used the MIG-HD trial (i) to assess whether the retest effect modified performance and whether our strategy of using the second assessment as a baseline was sensitive to cognitive decline in the long-term (A_3_) and (ii) to evaluate whether introducing the retest effect (ΔA_2_-A_1_) into the model of disease progression in patients improved the predictive value of the model in the long term (A_3_). Finally, we transferred the models obtained for the MIG-HD cohort to the RIL-HD cohort, to assess their predictive value in another population.

## Materials and Methods

### Participants and design

Patients were enrolled in two separate trials: the MIG-HD trial (*N* = 54, Ref NCT00190450, PI AC Bachoud-Lévi) [8], which is currently underway, and the placebo group of the cognitive ancillary study of the RIL-HD trial conducted only in France (*N* = 39, Ref NCT00277602, study coordinator Sanofi) [9]. Both trials were approved by the institutional review board (Comité Consultatif de Protection des Personnes dans la Recherche Biomédicale) of Henri-Mondor Hospital at Créteil (MIG-HD the September 25, 2001, and RIL-HD the December 18, 2002). Patients had signed an inform consent. The data were analyzed anonymously.

The MIG-HD trial is a phase II randomized trial assessing the efficacy of cell transplantation in HD patients at early stages of the disease. Patients were assessed at inclusion (A_1_), then 35 days (SD = 15) later (A_2_). They were randomized at one year (A_3_), to determine the timing of transplantation (M_13_-M_14_ for the early graft group or M_33_-M_34_ for the late graft group). Patients were followed up until 52 months.

The RIL-HD trial is a phase III multinational, randomized, placebo-controlled, double-blind study, for which a cognitive ancillary study was conducted in France from 1999 to 2004, on patients with moderately advanced HD. Patients were assessed at inclusion (A_1_), 15 days (SD = 8) later (A_2_) and at one year (A_3_), with randomization at A_2_.

The demographic features for patients at A_1_ are displayed in Table 1.

**Table 1:** Characteristics of patients at their inclusion (A_1_) in the MIG-HD and RIL-HD trials.

Characteristics	MIG-HD (*N* = 54)	RIL-HD (*N* = 39)
Age, y, mean (SD)	43.3 (8.7)	48.5 (10.1)
Sex % men / women	63.0 / 37.0	48.7 / 51.3
Education level, y, mean (SD)	12.0 (3.4)	12.3 (3.6)
Inheritance % paternal / maternal	60.0 / 40.0	47.6 / 52.4
Age of parent at onset, y, mean (SD)	42.2 (10.6)	45.7 (10.8)
Number of CAG repeats, mean (SD)	45.4 (4.2)	44.1 (3.6)
Time since onset, y, mean (SD)	4.5 (2.6)	6.1 (6.2)
TFC, mean (SD)	11.7 (1.0)	10.8 (1.8)
First symptom %		
Motor	60.7	70.3
Cognitive	17.9	13.5
Psychiatric	21.4	16.2

y: years; SD: standard deviation; TFC: total functional capacity.

### Clinical assessments

The Unified Huntington’s Disease Rating Scale (UHDRS) [10] and additional cognitive tests were used in both studies. Motor score reflected both voluntary and involuntary capacity and ranged from 0 to 124 (highest severity). Functional disability was assessed with Total Functional Capacity (TFC, range: 13 to 0) and Independence Scale (IS, range 100 to 0) scores, with lower scores indicating greater functional impairment, and the Functional Assessment Scale (FAS, 25 to 50), with higher scores indicating greater functional impairment. The severity and frequency of behavioral dysfunctions were quantified with the behavioral part of the UHDRS (range: 0 to 88), with higher scores indicating greater impairment. Global cognitive efficiency was evaluated with the Mattis Dementia Rating Scale (MDRS) [11]. Several tasks were used to assess attention and executive functions: letter fluency (for P, R and V in French) determined for 1 minute, the Symbol Digit Modalities Test (SDMT), the three components of the Stroop test (color naming, word reading, and color-word interference), each assessed for 45 seconds [12], categorical fluency (for animals) assessed for 1 minute [13],[14], the Trail-Making Test forms A and B (TMT A and B) [15], scoring the time taken to link 25 points, with a maximal time of 240 seconds, and figure cancellation tasks [16], in which patients were asked to cross out one, two and then three figures from a panel of signs, in 90 seconds, with lower scores indicating greater cognitive impairment. Short-term and long-term memory were evaluated with the Hopkins Verbal Learning Task (HVLT) including immediate recall, delayed recall and recognition tasks [17],[18]. By contrast to the other tasks, the HVLT was assessed with alternating parallel forms.

Each patient performed one motor test, three functional tests, one behavioral test and 15 cognitive tests at each assessment point.

### Statistical Analysis

#### Evaluation of the retest effect in the MIG-HD cohort

For each task, we used Student’s *t*-tests for paired data to compare performances, first between A_1_ and A_2_, to measure the potential retest effect, then between A_1_ and A_3,_ to assess the decline over a one-year period and between A_2_ and A_3_, to determine whether discarding the A_1_ data unmasked a decline that was otherwise undetectable.

#### Modeling of performance for the MIG-HD cohort

For each task, we selected the multivariate linear model best predicting the data at one year, by stepwise selection [19] with the Akaike Information Criterion (AIC) [20]. We used an iterative algorithm (stepwise selection) to select, without prior assumptions, the best predictive factors from a set of 10 variables (performance at A_1_, retest, age, sex, education level expressed as the number of years spent studying, parental inheritance, age of parent at disease onset, CAG repeat length, time since disease onset and the nature of the first symptom appearing at disease onset (motor, cognitive or psychiatric), as determined by the clinician or, if no clinician’s assessment was available, by the family or the patient). Lower AIC values indicate a better fit of the model to the data. The first model selection step was carried out for patients with complete data sets only. Estimates of regression coefficients were refined, by recalculating each model, using all the available complete data for the selected variables. The retest is the difference: performance at A_2_ –performance at A_1_ and is denoted ΔA_2_-A_1_. For each task, performance at A_3_ (*P*) was predicted as follows:

$$\begin{array}{l} P=\;\beta_{0}+\;\beta_{score\;at\;A_{1}}\times \;\text{performance at }A_{1}\;+\;\beta_{retest}\;\times \;\Delta {\text{A}}_{2}-{\text{A}}_{1}\;+\;\beta_{age\;at\;A_{1}}\times \;\text{age}\;\\ +\;\beta_{sex}\;+\;\beta_{education\;level}\times \;\text{education level}\;+\;\beta_{inheritance}\;+\;\beta_{age\;of\;parent\;at\;onset}\;\\ \times \;\text{age of parent at onset}+\;\beta_{CAG}\;\times \;\text{CAG}\;+\;\beta_{time\;since\;onset}\;\times \;\text{time since onset}\;\\ +\;\beta_{first\;symptom}\; \end{array}$$

where age, education level and age of parent at onset are expressed in years; the first symptom could be motor, cognitive or psychiatric; *β*_0_ is the intercept and, for each variable, *β*_*variable*_ is its associated regression coefficient (0 for the variables not selected). For quantitative variables, *β*_*variable*_ was multiplied by the value of the variable. For qualitative variables (sex, inheritance and first symptom), “woman”, “maternal inheritance” and “motor symptom” constituted the reference factors, such that *β*_*woman*_ = *β*_*maternal*_ = *β*_*motor*_ = 0. Calculation of the associated 95% predictive interval (95% PI) is explained in the supplemental data (S1 Text).

#### External validation on the RIL-HD cohort

We used models constructed from data for the MIG-HD cohort to predict performances at A_3_ for each patient in the RIL-HD cohort. Then, for each task, we measured the concordance between observed (*O*) and predicted (*P*) values, using the intraclass correlation coefficient (ICC) and the coefficient of determination ($R_{e}^{2}$). The ICC was calculated with a two-way mixed effect model [21] and evaluates agreement between observed (*O*) and predicted (*P*) performances at A_3_ in the RIL-HD cohort. The coefficient of determination ($R_{e}^{2}$) is the percentage of the observed performance variance explained by the model constructed from MIG-HD data. It assesses the degree to which observed performance at A_3_ in the RIL-HD cohort is accurately predicted by the model, as follows:

$$R_{e}^{2}=1-\frac{\underset{i}{\sum }\left(O_{i}-P_{i}\right)}{\underset{i}{\sum }\left(O_{i}-m\right)}$$

where *i* refers to a patient and *m* is the mean observed performance at A_3_. $R_{e}^{2}$ = 1 indicates a perfect predictive value of the model, whereas $R_{e}^{2}$ ≤ 0 indicates that the model is not informative.

Analyses were performed with R 2.13 software (http://www.r-project.org/). All tests were two-tailed and values of *P* < 0.05 were considered significant.

## Results

### Evaluating the retest effect in the MIG-HD cohort

**Fig 1:**
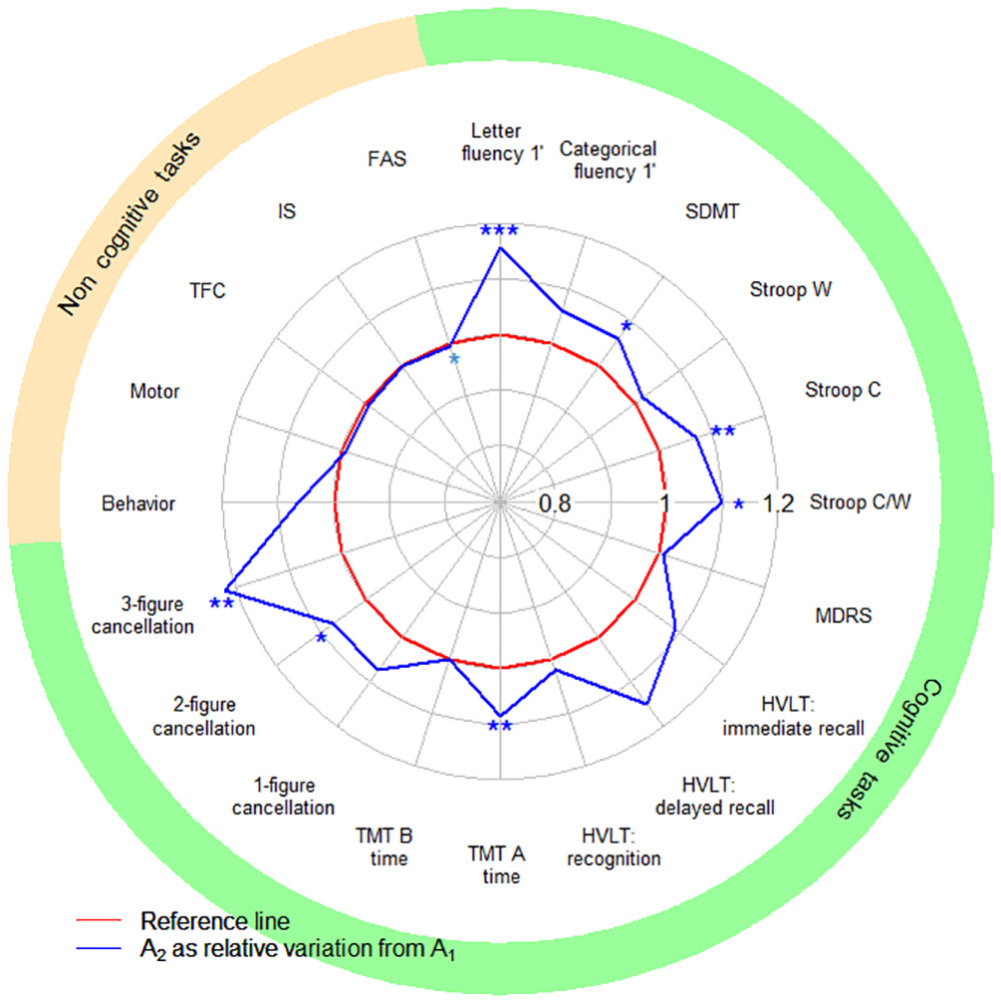
Impact of the retest effect in the MIG-HD cohort. SDMT: Symbol Digit Modalities Test; Stroop C, W and C/W: Stroop color, word and color/word interference; MDRS: Mattis Dementia Rating Scale; TMT A, B: Trail-Making Test A and B; TFC: Total Functional Capacity; IS: Independence Scale; FAS: Functional Assessment Scale. The red curve represents the baseline (reference score A_1_) and the blue curve shows the mean relative score one month later (A_2_). The portion of the blue curve beyond the red curve indicates performance improvement between A_1_ and A_2_. Paired *t*-tests, significance: * *P*<0.05, ** *P*<0.01, *** *P*<0.001.

We assessed the retest effect between A_1_ and A_2_ in the MIG-HD cohort. Performance improved in seven cognitive tasks, and remained stable in the other cognitive, motor and functional tasks, except for FAS score, which declined between A_1_ and A_2_ (Fig 1).

**Fig 2:**
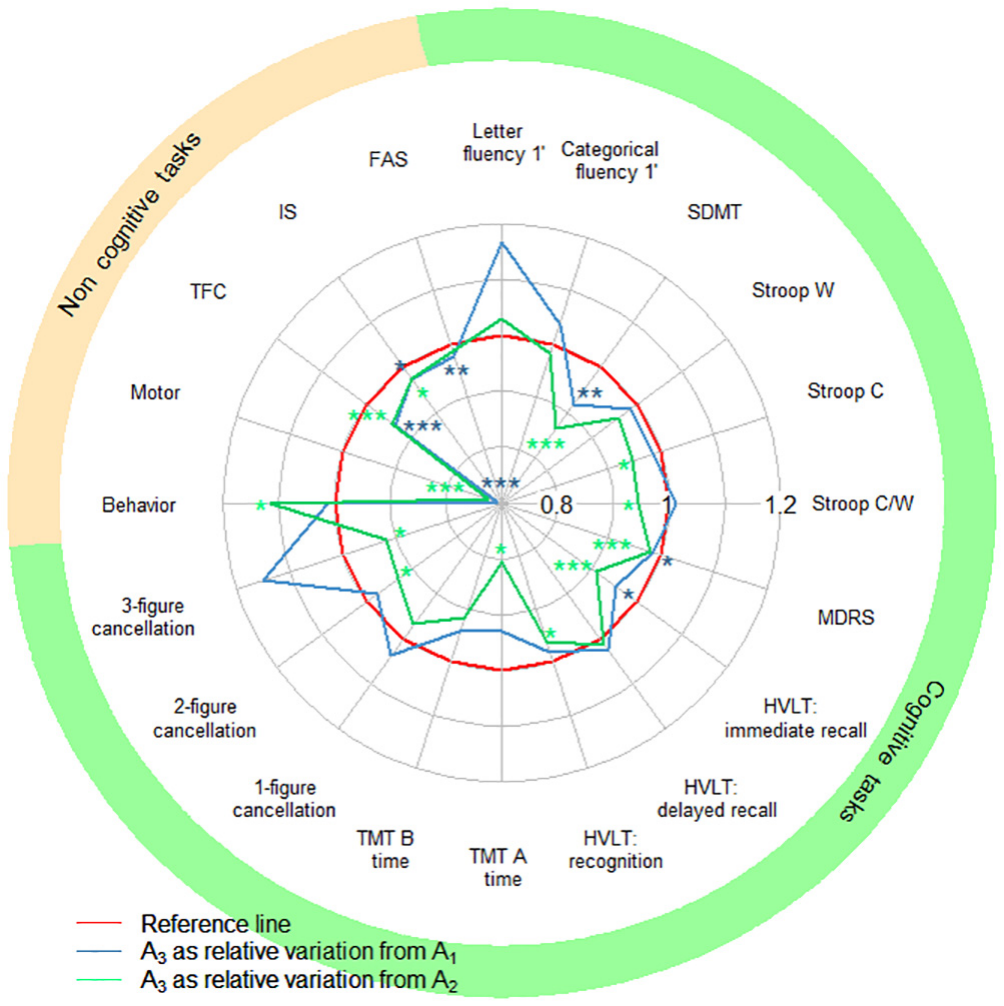
Observed performance at one year (A_3_), with A_1_ or A_2_ used as the baseline, in the MIG-HD cohort. SDMT: Symbol Digit Modalities Test; Stroop C, W and C/W: Stroop color, word and color/word interference; MDRS: Mattis Dementia Rating Scale; TMT A, B: Trail-Making Test A and B; TFC: Total Functional Capacity; IS: Independence Scale; FAS: Functional Assessment Scale. The red curve represents the baseline (reference score). The blue (or green) curve corresponds to the mean relative score one year later (A_3_), with A_1_ (or A_2_ for the green curve) used as the baseline. A green curve within the blue curve indicates that the decline was easier to detect if A_2_ was used as the baseline, rather than A_1_. Paired *t*-tests, significance: * *P*<0.05, ** *P*<0.01, *** *P*<0.001.

We assessed decline between A_1_ and A_3_ and between A_2_ and A_3_ in the MIG-HD cohort (Fig 2). The use of A_2_ as the baseline increased the number of tasks for which a decline in performance was detected from three to nine, but FAS score was the only motor or functional performance affected. Indeed, FAS performance declined between A_1_ and A_3_ but not between A_2_ and A_3_. Behavioral performance improved between A_2_ and A_3_.

### Modeling of performance in the MIG-HD cohort

Table 2 displays the regression coefficients of the predictive model for each task, for the MIG-HD cohort. Performance at A_1_ was predictive of performance at A_3_ in all tasks. Introducing the difference in performance between A_1_ and A_2_ (ΔA_2_-A_1_) into the models improved the prediction of performance at A_3_ for 14 of the 15 cognitive tasks, for behavioral and motor performance and TFC. Larger numbers of CAG repeats were associated with a poorer FAS and IS scale scores and poorer motor performance, but better behavioral performance. Women outperformed men in 7 of the 15 cognitive tasks. Sex had no effect on motor and functional performances, whereas behavioral performance was better in women than in men. Higher education levels were associated with better performance at A_3_ for all components of the HVLT.

**Table 2:** Predictive factors for each task.

		β_0_	β_score at A1_	β_retest_	β_age at A1_	Β_sex = man_	Β_education level_	β_inheritance = paternal_	β_age of parent at onset_	β_CAG_	Β_time since onset_	β_first symptom = cognitive_	β_first symptom = psychiatric_
**Cognitive**	**Letter Fluency 1’**	10.27* (3.92)	0.66*** (0.14)	0.84*** (0.18)		-2.55 (2.51)							
	**Categorical Fluency 1’**	6.55** (1.98)	0.57*** (0.13)	0.55*** (0.15)		-1.82 (0.93)							
	**SDMT**	-0.84 (2.19)	0.98*** (0.07)	0.33* (0.15)		-1.93 (1.22)							
	**Stroop W**	1.56 (8.67)	0.93*** (0.13)	1.04*** (0.22)									
	**Stroop C**	3.03 (5.66)	1.01*** (0.10)	0.43* (0.18)		-8.43** (2.58)							
	**Stroop W/C**	2.07 (3.17)	0.97*** (0.10)	0.65*** (0.14)		-3.65* (1.64)							
	**HVLT: Immediate recall**	6.08 (3.07)	0.53*** (0.10)	0.27* (0.12)		-2.01 (1.11)	0.26 (0.17)					-2.08 (1.37)	0.45 (1.22)
	**HVLT: delayed recall**	-0.51 (1.18)	0.55*** (0.10)				0.23* (0.09)						
	**HVLT: recognition**	-1.35 (1.60)	0.87*** (0.14)	0.52*** (0.13)			0.19** (0.06)						
	**MDRS**	20.29 (13.26)	0.89*** (0.09)	0.64*** (0.14)	-0.10 (0.07)	-2.81* (1.25)		-1.12 (1.22)			-0.39 (0.25)	0.18 (1.63)	1.97 (1.32)
	**1-figure cancellation**	1.51 (1.54)	0.89*** (0.08)	0.57*** (0.14)									
	**2-figure cancellation**	0.24 (1.55)	0.93*** (0.08)	0.50** (0.16)									
	**3-figure cancellation**	7.28* (2.74)	0.83*** (0.11)	0.55* (0.20)									
	**TMT A time**	13.11 (10.81)	0.90*** (0.15)	0.59** (0.18)									
	**TMT B time**	28.68* (22.40)	0.94*** (0.06)	0.86*** (0.11)	-0.98 (0.56)				0.63 (0.44)				
	**Behavior**	40.26** (13.96)	0.31* (0.12)	0.52** (0.16)		2.91 (2.08)	-0.92** (0.32)			-0.68* (0.28)	1.36** (0.48)		
	**Motor**	-32.91 (26.47)	0.81*** (0.1)	0.68** (0.21)	0.52* (0.25)			4.40 (2.67)	-0.23 (0.14)	0.63 (0.43)			
**Functional**	**FAS**	0.42 (5.44)	0.65*** (0.14)		0.03 (0.04)					0.18* (0.07)			
	**IS**	70.41* (28.28)	0.63*** (0.16)		-0.22 (0.19)					-0.66 (0.38)			
	**TFC**	-0.55 (2.18)	0.98*** (0.19)	1.50*** (0.4)	-0.003 (0.02)			0.35 (0.34)					

SDMT: Symbol Digit Modalities Test; Stroop C, W and C/W: Stroop color, word and color/word interference; HVLT: Hopkins Verbal Learning Task; MDRS: Mattis Dementia Rating Scale; TMT A, B: Trail-Making Test A and B; FAS: Functional Assessment Scale; IS: Independence Scale; TFC: Total Functional Capacity. A given row shows the predictive factors (estimated regression coefficient, standard error and significance: * *P*<0.05, ** *P*<0.01, *** *P*<0.001) for the corresponding task. The absence of a value indicates that the covariate concerned was not selected for the model.

The regression coefficients presented in Table 2 are those used in the predictive models. For example, the performance at A_3_ in letter Fluency 1’ is given by the following formula:

$$\text{performance at }A_{3}=\{\begin{array}{c} 10.27+0.66\;\times \text{performance at }A_{1}+0.84\;\times \text{retest}\;\;\;\;\;\;\;\text{woman}\\ 10.27+0.66\;\times \text{performance at }A_{1}+0.84\;\times \text{retest}-2.55\;\text{man} \end{array}$$

The equations associated with the predictive models for each task are detailed in S1 Table. Moreover, S2 Table gives additional parameters for calculation of the 95% PI.

### External validation on the RIL-HD cohort

**Fig 3:**
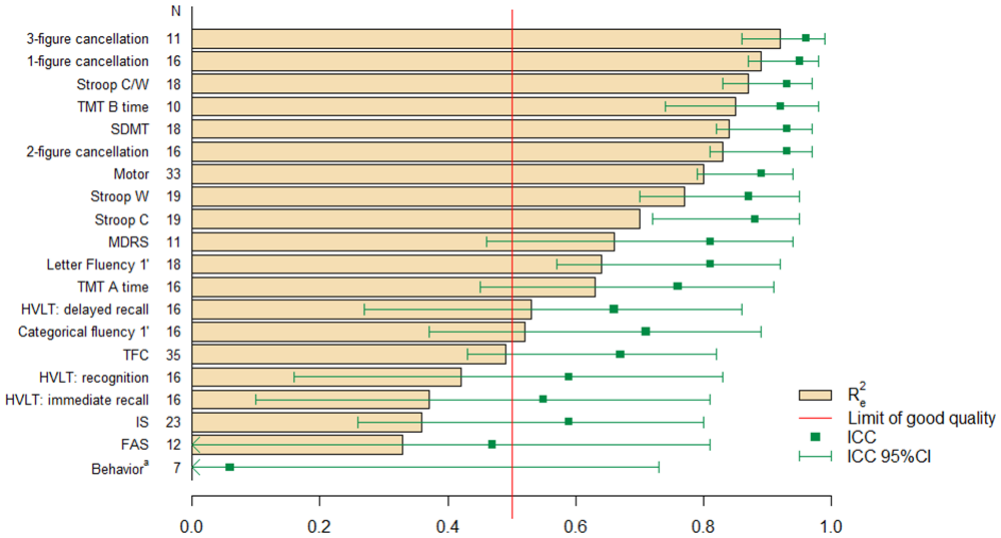
External validation of models in the RIL-HD cohort, based on *R*_*e*_^*2*^ and ICC. SDMT: Symbol Digit Modalities Test; Stroop C, W and C/W: Stroop color, word and color/word interference; MDRS: Mattis Dementia Rating Scale; TMT A, B: Trail-Making Test A and B; HVLT: Hopkins Verbal Learning Task; TFC: Total Functional Capacity; IS: Independence Scale; FAS: Functional Assessment Scale. N: number of patients in the RIL-HD cohort for whom all the data required for the predictive model were available. *R*_*e*_^*2*^: coefficient of determination for external validation. ICC: intraclass correlation coefficient. 95% CI: 95% confidence interval. a: *R*_*e*_^*2*^ = -0.7. The red line represents the limit for a high-quality model (*R*_*e*_^*2*^ > 50% of the observed variance explained by the model).

For each task, we determined the predictive value of models by calculating the ICC and $R_{e}^{2}$ (Fig 3). Performance in the RIL-HD trial was well predicted for 14 of 20 tasks by the models developed with data for the MIG-HD cohort ($R_{e}^{2}$ ≥ 0.5 and ICC ≥ 0.6).

## Discussion

The design of clinical trials for neurodegenerative diseases could be improved by methodological approaches based on our knowledge of the patient’s cognitive performances. However, cognitive knowledge is obtained mostly through longitudinal follow-up in observational studies, which may not include variability factors inherent to clinical trials. The retest effect may impede observations of cognitive decline in patients with Huntington’s disease. We therefore assessed its impact in two long-term clinical trials in HD patients, with a short interval between the first and second assessments (MIG-HD, RILH-HD). We first determined whether there was a detectable retest effect between the first two assessments (A_1_ and A_2_), and then evaluated the impact of this effect one year later (A_3_). We found that replacing A_1_ with A_2_ as the baseline unmasked a decline that would not otherwise have been detected. Indeed, the comparison between A_2_ and A_3_ showed declines that were not apparent in the comparison between A_1_ and A_3_. We also modeled patient performance and showed how the inclusion of the retest effect in patient performance models would improve trial design.

At one year, decline was observed in a few cognitive tasks (SDMT, MDRS and the HVLT immediate recall), the motor task and all functional tasks. However, consistent with previous findings [2], there was a pronounced retest effect in cognitive tasks (letter fluency, SDMT, Stroop color and color/word interference, TMT A and 2- and 3-figure cancellation tasks), but no such effect in motor and functional assessments. This retest effect may hamper the objective detection of cognitive decline, with a major impact in tasks with a high cognitive demand, obscuring performance decline over a one-year period [22]. Neutralization of the retest effect is particularly important in clinical trials, because some patients may already have been exposed to testing whereas others have not, adding background noise to the overall performance data. Assuming that the retest effect is maximal at the second assessment, the use of this assessment as the baseline can decrease the impact of the retest effect on subsequent assessments. By discarding performances at A_1_ and using the performance measured at A_2_ as the baseline, we unmasked a decline in six tasks (Stroop color and color/word interference, recognition part of HVLT, TMT A and 2- and 3-figure cancellation), demonstrating the efficacy of this strategy for small samples. However, the improvement in behavioral performance [23], contrasting with the decline in other task performances, may reflect the patients’ hopes and expectations of treatment.

The HVLT constitutes a specific case: we alternated parallel forms because of the strength of item recall in declarative memory tasks [24]. However, alternation was not used for other tasks, because parallel forms are of no interest for procedural tasks or tasks with a strong motor output (SDMT, TMT A and verbal fluency tasks) [25]. The use of parallel forms should also be limited because of their low intrasubject equivalence, potentially introducing noise into longitudinal performance analyses. Furthermore, the ceiling effect observed in patients with high scores in the HVLT, MDRS and TMT tasks limits the utility of neutralizing the retest effect.

However, the retest effect depends not only on the nature of the task, but also on the population assessed [26]. Indeed, Cooper *et al*. [27], [28] demonstrated the existence of a retest effect in categorical fluency assessment in healthy participants but not in patients with Alzheimer’s disease or mild cognitive impairments. Likewise, we found no retest effect for this task in HD patients.

In addition to masking decline, the retest effect may provide information about disease progression [7]. This suggests that combining a strategy based on the individual performance of patients and the nature of the tasks may be useful. Indeed, the modeling of patient performance at one year for each task showed that ΔA_2_-A_1_ performance, even in the absence of a significant retest effect, accurately predicted performance for most cognitive tasks in HD and for motor and behavior tasks and TFC. ΔA_2_-A_1_ performance appears to be more frequently selected by stepwise algorithms than sociodemographic and genetic variables. We also arbitrated between parameters to strengthen our models. For example, both the number of CAG repeats and age at onset are eligible variables [29], but they are correlated [30–32], so only one of these factors should be included in the model [33]. We decided to include the number of CAG repeats, as age at onset is subject to some degree of subjectivity. Likewise, rather than using the performance in one task to explain performance in another task (e.g. using motor score to explain TFC [34]), we limited the set of eligible variables to demographic variables. Finally, we did not include handedness in our models, because 90% of the patients were right-handed.

This approach made it possible to include a larger number of covariates in our models than in those of previous studies and to prioritize them through the selection algorithm. For example, the number of CAG repeats has been reported to affect general verbal and spatial abilities [35], whereas our stepwise selection suggested that it was predictive of performance in the 3-figure cancellation task, which has a spatial nonverbal component. Indeed, the number of CAG repeats was found to have less impact than the sex of the patient in verbal tasks (letter and categorical fluencies) and sex was not included in the model described in the previous study. Furthermore, dichotomization of the number of CAG repeats variable (small and large numbers of repeats) may have resulted in greater importance being assigned to this variable than in models, such as ours, in which the number of CAG repeats was treated as a continuous variable. Like Ruocco [36], Kieburtz [37] and Feigin *et al*. [38], we showed that the number of CAG repeats improved in the prediction of motor performance, but not TFC. Finally, higher education levels were associated with a better performance, for all HVLT components.

The small number of patients enrolled in the MIG-HD study is a potential limitation in the search for predictive factors for future studies. However, external validation on the RIL-HD cohort, through calculation of the intraclass correlation coefficient and the determination coefficient ($R_{e}^{2}$), demonstrated the reproducibility and robustness of our models, regardless of the differences between the two trials. Indeed, patients in the MIG-HD trial were not randomized until one year (A_3_), whereas those in the RIL-HD study were randomized at the second assessment (A_2_). Consequently, the patients in the MIG-HD study approached the intervention with greater hope, whereas those in the placebo group of the RIL-HD study may have been aware of a lack of improvement during the follow-up period. This difference may account for the poor prediction of behavioral performance in the RIL-HD study ($R_{e}^{2}$ < 0). By contrast, the difference in time interval between A_1_ and A_2_ in the two studies had no impact on prediction quality, further demonstrating the validity of the models. The models were constructed with data from patients with relatively mild disease. They may, therefore, not be applicable to patients with more advanced HD. Indeed, retest effects would be expected to be smaller in patients with more severe disease.

Our findings indicate that the retest effect is a limitation in clinical trials, but that both its neutralization, through the use of a second assessment as a baseline, and its integration into task modeling would be beneficial in future trials. For example, our predictive models may facilitate the identification of rapid decliners [39], defined as individuals whose observed performance is worse than predicted. Indeed, in longitudinal clinical trials, treatment effects could be masked in such patients, as already shown for Alzheimer’s disease [40]. The identification of such patients is helpful for trial design, in two ways. First, the exclusion of such patients would probably decrease intersubject variability, making it possible to decrease sample size. Second, rapid decliners could be uniformly allocated to the different arms of the study by stratified randomization, to ensure the constitution of comparable groups, in terms of both baseline data and disease progression.

Our findings suggest that the retest effect is detrimental, if uncontrolled, in clinical trials for neurodegenerative diseases, such as Huntington’s disease. We show here that if two assessments are performed a short time apart, use of the second assessment as the baseline increases the chances of detecting an effect of treatment, if there is one. In addition, including the retest effect in models renders the resulting models more predictive, making it possible to refine the design of future trials. This constitutes a great stride forward in cognitive assessments in clinical trials.

## Supporting Information

S1 Table Predictive model for each task. (DOCX)

S2 Table M matrix for calculating the 95% prediction interval for performance at A_3_ for each task. (DOCX)

S1 Text Statistical explanation for calculation of the 95% prediction interval for performance at A_3_, for each task. (DOCX)
