# Supplementary material for: How to Capitalize on the Retest Effect in Future Trials on Huntington’s Disease
Source: PLoS One. 2015 Dec 29;10(12):e0145842. doi: 10.1371/journal.pone.0145842 (PMC4703129; doi:10.1371/journal.pone.0145842)
Supplement: S1 Table — (DOCX) [file pone.0145842.s001.docx]

**S1 Table. Predictive model for each task**

| **Task** | **Model** |
| --- | --- |
| Letter Fluency 1’ | Women: $10.27+0.66\times\text{score at }A_{1}+0.84 \times\text{retest}$  Men: $10.27+0.66\times\text{score at }A_{1}+0.84 \times\text{retest}-2.55$ |
| Categorical Fluency 1’ | Women: $6.55+0.57\times\text{score at }A_{1}+0.55 \times\text{retest}$  Men: $6.55+0.57\times\text{score at }A_{1}+0.55 \times\text{retest}-1.82$ |
| SDMT | Women: $-0.84+0.98\times\text{score at }A_{1}+0.33 \times\text{retest}$  Men: $-0.84+0.98\times\text{score at }A_{1}+0.33 \times\text{retest}-1.93$ |
| Stroop W | $1.56+0.93\times\text{score at }A_{1}+1.04 \times\text{retest}$ |
| Stroop C | Women: $3.03+1.01\times\text{score at }A_{1}+0.43 \times\text{retest}$  Men: $3.03+1.01\times\text{score at }A_{1}+0.43 \times\text{retest}-8.43$ |
| Stroop C/W | Women: $2.07+0.97\times\text{score at }A_{1}+0.65 \times\text{retest}$  Men: $2.07+0.97\times\text{score at }A_{1}+0.65 \times\text{retest}-3.65$ |
| HVLT: Immediate recall | Women and motor first symptom: $6.08+0.53\times\text{score at }A_{1}+0.27 \times\text{retest}+0.26\times\text{education level}$  Women and cognitive first symptom: $6.08+0.53\times\text{score at }A_{1}+0.27 \times\text{retest}+0.26\times\text{education level}-2.08$  Women and psychiatric first symptom: $6.08+0.53\times\text{score at }A_{1}+0.27 \times\text{retest}+0.26\times\text{education level}+0.45$  Men and motor first symptom: $6.08+0.53\times\text{score at }A_{1}+0.27 \times\text{retest}-2.01+0.26\times\text{education level}$  Men and cognitive first symptom: $6.08+0.53\times\text{score at }A_{1}+0.27 \times\text{retest}-2.01+0.26\times\text{education level}-2.08$  Men and psychiatric first symptom: $6.08+0.53\times\text{score at }A_{1}+0.27 \times\text{retest}-2.01+0.26\times\text{education level}+0.45$ |
| HVLT: delayed recall | $-0.51+0.55\times\text{score at }A_{1}+0.23\times\text{education level}$ |
| HVLT recognition | $-1.35+0.87\times\text{score at }A_{1}+0.52\times\text{retest}+0.19\times\text{education level}$ |
| MDRS | Women, motor first symptom and maternal inheritance: $20.29+0.89\times\text{score at }A_{1}+0.64 \times\text{retest}-0.10\times\text{age at }A_{1}-0.39\times\text{time since onset}$  Women, motor first symptom and paternal inheritance: $0.29+0.89\times\text{score at }A_{1}+0.64 \times\text{retest}-0.10\times\text{age at }A_{1}-1.12-0.39\times\text{time since onset}$  Women, cognitive first symptom and maternal inheritance: $20.29+0.89\times\text{score at }A_{1}+0.64 \times\text{retest}-0.10\times\text{age at }A_{1}-0.39\times\text{time since onset}+0.18$  Women, cognitive first symptom and paternal inheritance: $0.29+0.89\times\text{score at }A_{1}+0.64 \times\text{retest}-0.10\times\text{age at }A_{1}-1.12-0.39\times\text{time since onset}+0.18$  Women, psychiatric first symptom and maternal inheritance: $20.29+0.89\times\text{score at }A_{1}+0.64 \times\text{retest}-0.10\times\text{age at }A_{1}-0.39\times\text{time since onset}+1.97$  Women, psychiatric first symptom and paternal inheritance: $0.29+0.89\times\text{score at }A_{1}+0.64 \times\text{retest}-0.10\times\text{age at }A_{1}-1.12-0.39\times\text{time since onset}+1.97$  Men, motor first symptom and maternal inheritance: $20.29+0.89\times\text{score at }A_{1}+0.64 \times\text{retest}-0.10\times\text{age at }A_{1}-2.81-0.39\times\text{time since onset}$  Men, motor first symptom and paternal inheritance: $0.29+0.89\times\text{score at }A_{1}+0.64 \times\text{retest}-0.10\times\text{age at }A_{1}-2.81-1.12-0.39\times\text{time since onset}$  Men, cognitive first symptom and maternal inheritance: $20.29+0.89\times\text{score at }A_{1}+0.64 \times\text{retest}-0.10\times\text{age at }A_{1}-2.81-0.39\times\text{time since onset}+0.18$  Men, cognitive first symptom and paternal inheritance: $0.29+0.89\times\text{score at }A_{1}+0.64 \times\text{retest}-0.10\times\text{age at }A_{1}-2.81-1.12-0.39\times\text{time since onset}+0.18$  Men, psychiatric first symptom and maternal inheritance: $20.29+0.89\times\text{score at }A_{1}+0.64 \times\text{retest}-0.10\times\text{age at }A_{1}-2.81-0.39\times\text{time since onset}+1.97$  Men, psychiatric first symptom and paternal inheritance: $0.29+0.89\times\text{score at }A_{1}+0.64 \times\text{retest}-0.10\times\text{age at }A_{1}-2.81-1.12-0.39\times\text{time since onset}+1.97$ |
| 1-figure cancellation | $1.51+0.89\times\text{score at }A_{1}+0.57\times\text{retest}$ |
| 2-figure cancellation | $0.24+0.93\times\text{score at }A_{1}+0.50\times\text{retest}$ |
| 3-figure cancellation | Maternal inheritance: $7.28+0.83\times\text{score at }A_{1}+0.55\times\text{retest}+0.11\times\text{age of parent at onset}$  Paternal inheritance: $7.28+0.83\times\text{score at }A_{1}+0.55\times\text{retest}-1.96+0.11\times\text{age of parent at onset}$ |
| TMT A time | $13.11+0.90\times\text{score at }A_{1}+0.59\times\text{retest}$ |
| TMT B time | $28.68+0.94\times\text{score at }A_{1}+0.86\times\text{retest}-0.98\times\text{age at }A_{1}+0.63\times\text{age of parent at onset}$ |
| Behavior | Women: $40.26+0.31\times\text{score at }A_{1}+0.52\times\text{retest}-0.92\times\text{education level}-0.68\times\text{CAG}+1.36\times\text{time since onset}$  Men: $40.26+0.31\times\text{score at }A_{1}+0.52\times\text{retest}+2.91-0.92\times\text{education level}-0.68\times\text{CAG}+1.36\times\text{time since onset}$ |
| Motor | Maternal inheritance: $-32.91+0.81\times\text{score at }A_{1}+0.68\times\text{retest}+0.52\times\text{age at }A_{1}-0.23\times\text{age of parent at onset}+0.63\times\text{CAG}$  Paternal inheritance: $-32.91+0.81\times\text{score at }A_{1}+0.68\times\text{retest}+0.52\times\text{age at }A_{1}+4.40-0.23\times\text{age of parent at onset}+0.63\times\text{CAG}$ |
| FAS | $0.42+0.65\times\text{score at }A_{1}+0.03\times\text{age at }A_{1}+0.18\times\text{CAG}$ |
| IS | $70.41+0.63\times\text{score at }A_{1}-0.22\times\text{age at }A_{1}-0.66\times\text{CAG}$ |
| TFC | Maternal inheritance: $-0.55+0.98\times\text{score at }A_{1}+1.50\times\text{retest}-0.003\times\text{age at }A_{1}$  Paternal inheritance: $-0.55+0.98\times\text{score at }A_{1}+1.50\times\text{retest}-0.003\times\text{age at }A_{1}+0.35$ |

SDMT: Symbol Digit Modalities Test; Stroop C, W and C/W: Stroop color, word and color/word interference; HVLT: Hopkins Verbal Learning Task; MDRS: Mattis Dementia Rating Scale; TMT A, B: Trail-Making Test A and B; FAS: Functional Assessment Scale; IS: Independence Scale; TFC: Total Functional Capacity.
