# Supplementary material for: How to Capitalize on the Retest Effect in Future Trials on Huntington’s Disease
Source: PLoS One. 2015 Dec 29;10(12):e0145842. doi: 10.1371/journal.pone.0145842 (PMC4703129; doi:10.1371/journal.pone.0145842)
Supplement: S2 Table — (DOCX) [file pone.0145842.s002.docx]

**S2 Table. M matrix for calculating the 95% prediction interval for performance at A_3_ for each task**

| k  l | | $\hat{\sigma}$ | Intercept | Score  at A_1_ | Retest  (ΔA_2_-A_1_) | Age  at A_1_ | Sex | Education  level | Inheritance | Age of  parent  at onset | CAG | Time since onset | First symptom | |
| --- | --- | --- | --- | --- | --- | --- | --- | --- | --- | --- | --- | --- | --- | --- |
|  |  |  |  |  |  |  |  |  |  |  |  |  | Cognitive | Psychiatric |
| **Letter fluency 1’** | | 7.786 |  |  |  |  |  |  |  |  |  |  |  |  |
| Intercept | |  | 0.25358 |  |  |  |  |  |  |  |  |  |  |  |
| Score at A_1_ | |  | -0.00754 | 0.00031 |  |  |  |  |  |  |  |  |  |  |
| Retest (ΔA_2_-A_1_) | |  | -0.00214 | -0.00001 | 0.00054 |  |  |  |  |  |  |  |  |  |
| Sex | |  | -0.04983 | -0.00074 | 0.00123 |  | 0.10401 |  |  |  |  |  |  |  |
| **Categorical fluency 1’** | | 2.915 |  |  |  |  |  |  |  |  |  |  |  |  |
| Intercept | |  | 0.45934 |  |  |  |  |  |  |  |  |  |  |  |
| Score at A_1_ | |  | -0.02805 | 0.00199 |  |  |  |  |  |  |  |  |  |  |
| Retest (ΔA_2_-A_1_) | |  | -0.01490 | 0.00094 | 0.00256 |  |  |  |  |  |  |  |  |  |
| Sex | |  | -0.07094 | 0.00059 | 0.00048 |  | 0.10115 |  |  |  |  |  |  |  |
| **SDMT** | | 3.835 |  |  |  |  |  |  |  |  |  |  |  |  |
| Intercept | |  | 0.32575 |  |  |  |  |  |  |  |  |  |  |  |
| Score at A_1_ | |  | -0.00969 | 0.00037 |  |  |  |  |  |  |  |  |  |  |
| Retest (ΔA_2_-A_1_) | |  | -0.00438 | 0.00003 | 0.00161 |  |  |  |  |  |  |  |  |  |
| Sex | |  | -0.06106 | -0.00018 | 0.00138 |  | 0.10083 |  |  |  |  |  |  |  |
| **Stroop C** | | 11.84 |  |  |  |  |  |  |  |  |  |  |  |  |
| Intercept | |  | 0.60485 |  |  |  |  |  |  |  |  |  |  |  |
| Score at A_1_ | |  | -0.00796 | 0.00012 |  |  |  |  |  |  |  |  |  |  |
| Retest (ΔA_2_-A_1_) | |  | -0.00453 | 0.00006 | 0.00034 |  |  |  |  |  |  |  |  |  |
| Sex | |  | -0.08288 | 0.00030 | 0.00005 |  | 0.10035 |  |  |  |  |  |  |  |
| **Stroop W** | | 8.146 |  |  |  |  |  |  |  |  |  |  |  |  |
| Intercept | |  | 0.51270 |  |  |  |  |  |  |  |  |  |  |  |
| Score at A_1_ | |  | -0.00985 | 0.00040 |  |  |  |  |  |  |  |  |  |  |
| Retest (ΔA_2_-A_1_) | |  | -0.00465 | 0.00008 | 0.00048 |  |  |  |  |  |  |  |  |  |
| **Stroop C/W** | | 5.372 |  |  |  |  |  |  |  |  |  |  |  |  |
| Intercept | |  | 0.3831 |  |  |  |  |  |  |  |  |  |  |  |
| Score at A_1_ | |  | -0.01078 | 0.00037 |  |  |  |  |  |  |  |  |  |  |
| Retest (ΔA_2_-A_1_) | |  | -0.00458 | 0.00010 | 0.00071 |  |  |  |  |  |  |  |  |  |
| Age at A_1_ | |  |  |  |  |  |  |  |  |  |  |  |  |  |
| Sex | |  | -0.08905 | 0.00085 | 0.00091 |  | 0.10216 |  |  |  |  |  |  |  |
| **HVLT: immediate recall** | | 3.183 |  |  |  |  |  |  |  |  |  |  |  |  |
| Intercept | |  | 0.93121 |  |  |  |  |  |  |  |  |  |  |  |
| Score at A_1_ | |  | -0.02234 | 0.00108 |  |  |  |  |  |  |  |  |  |  |
| Retest (ΔA_2_-A_1_) | |  | -0.01596 | 0.00048 | 0.00139 |  |  |  |  |  |  |  |  |  |
| Sex | |  | -0.13675 | 0.00340 | 0.00047 |  | 0.12118 |  |  |  |  |  |  |  |
| Education level | |  | -0.02796 | -0.00018 | 0.00045 |  | -0.00074 | 0.00271 |  |  |  |  |  |  |
| First symptom | Cognitive |  | -0.00787 | 0.00011 | -0.00262 |  | 0.01535 | -0.00428 |  |  |  |  | 0.18405 |  |
|  | Psychiatric |  | 0.03215 | -0.00163 | -0.00271 |  | -0.01555 | -0.00299 |  |  |  |  | 0.05352 | 0.14796 |
| **HVLT: delayed recall** | | 1.783 |  |  |  |  |  |  |  |  |  |  |  |  |
| Intercept | |  | 0.43801 |  |  |  |  |  |  |  |  |  |  |  |
| Score at A_1_ | |  | -0.01399 | 0.00297 |  |  |  |  |  |  |  |  |  |  |
| Education level | |  | -0.02571 | -0.00055 |  |  |  | 0.00240 |  |  |  |  |  |  |
| **HVLT: recognition** | | 1.144 |  |  |  |  |  |  |  |  |  |  |  |  |
| Intercept | |  | 1.94602 |  |  |  |  |  |  |  |  |  |  |  |
| Score at A_1_ | |  | -0.15123 | 0.01467 |  |  |  |  |  |  |  |  |  |  |
| Retest (ΔA_2_-A_1_) | |  | -0.07514 | 0.00614 | 0.01207 |  |  |  |  |  |  |  |  |  |
| Education level | |  | -0.02551 | -0.00037 | 0.00066 |  |  | 0.00238 |  |  |  |  |  |  |
| **MDRS** | | 3.463 |  |  |  |  |  |  |  |  |  |  |  |  |
| Intercept | |  | 14.67372 |  |  |  |  |  |  |  |  |  |  |  |
| Score at A_1_ | |  | -0.10020 | 0.00074 |  |  |  |  |  |  |  |  |  |  |
| Retest (ΔA_2_-A_1_) | |  | -0.06547 | 0.00044 | 0.00169 |  |  |  |  |  |  |  |  |  |
| Age at A_1_ | |  | -0.01846 | 0.000004 | 0.00006 | 0.00040 |  |  |  |  |  |  |  |  |
| Sex | |  | -0.31087 | 0.00213 | 0.00351 | -0.00122 | 0.12977 |  |  |  |  |  |  |  |
| Inheritance | |  | -0.14897 | 0.000002 | 0.00121 | 0.00183 | -0.01876 |  | 0.12425 |  |  |  |  |  |
| Time since onset | |  | -0.05450 | 0.00021 | 0.00045 | 0.00005 | 0.00300 |  | -0.00021 |  |  | 0.00502 |  |  |
| First symptom | Cognitive |  | -0.09605 | 0.00067 | -0.00613 | -0.00043 | -0.00234 |  | -0.01223 |  |  | -0.00303 | 0.22221 |  |
|  | Psychiatric |  | -0.05031 | -0.00016 | -0.00145 | 0.00076 | -0.02013 |  | 0.01822 |  |  | -0.00180 | 0.05246 | 0.14541 |
| **1-figure cancellation** | | 2.786 |  |  |  |  |  |  |  |  |  |  |  |  |
| Intercept | |  | 0.30624 |  |  |  |  |  |  |  |  |  |  |  |
| Score at A_1_ | |  | -0.01580 | 0.00090 |  |  |  |  |  |  |  |  |  |  |
| Retest (ΔA_2_-A_1_) | |  | -0.01158 | 0.00053 | 0.00249 |  |  |  |  |  |  |  |  |  |
| **2-figure cancellation** | | 2.958 |  |  |  |  |  |  |  |  |  |  |  |  |
| Intercept | |  | 0.27298 |  |  |  |  |  |  |  |  |  |  |  |
| Score at A_1_ | |  | -0.01401 | 0.00080 |  |  |  |  |  |  |  |  |  |  |
| Retest (ΔA_2_-A_1_) | |  | -0.00707 | 0.00028 | 0.00310 |  |  |  |  |  |  |  |  |  |
| **3-figure cancellation** | | 3.120 |  |  |  |  |  |  |  |  |  |  |  |  |
| Intercept | |  | 0.77306 |  |  |  |  |  |  |  |  |  |  |  |
| Score at A_1_ | |  | -0.01394 | 0.00134 |  |  |  |  |  |  |  |  |  |  |
| Retest (ΔA_2_-A_1_) | |  | -0.02077 | 0.00112 | 0.00413 |  |  |  |  |  |  |  |  |  |
| Inheritance | |  | -0.09728 | 0.00040 | 0.00459 |  |  |  | 0.14470 |  |  |  |  |  |
| Age of parent at onset | |  | -0.01095 | -0.00015 | -0.00011 |  |  |  | -0.00008 | 0.00032 |  |  |  |  |
| **TMT A time** | | 23.42 |  |  |  |  |  |  |  |  |  |  |  |  |
| Intercept | |  | 0.21318 |  |  |  |  |  |  |  |  |  |  |  |
| Score at A_1_ | |  | -0.00272 | 0.00004 |  |  |  |  |  |  |  |  |  |  |
| Retest (ΔA_2_-A_1_) | |  | -0.00115 | 0.00002 | 0.00006 |  |  |  |  |  |  |  |  |  |
| **TMT B time** | | 22.85 |  |  |  |  |  |  |  |  |  |  |  |  |
| Intercept | |  | 0.96053 |  |  |  |  |  |  |  |  |  |  |  |
| Score at A_1_ | |  | -0.00072 | 0.00001 |  |  |  |  |  |  |  |  |  |  |
| Retest (ΔA_2_-A_1_) | |  | -0.00058 | 0.000002 | 0.00003 |  |  |  |  |  |  |  |  |  |
| Age at A_1_ | |  | -0.01293 | -0.00002 | -0.00001 | 0.00059 |  |  |  |  |  |  |  |  |
| Age of parent at onset | |  | -0.00681 | 0.00001 | 0.00001 | -0.00024 |  |  |  | 0.00038 |  |  |  |  |
| **Motor** | | 7.213 |  |  |  |  |  |  |  |  |  |  |  |  |
| Intercept | |  | 13.46612 |  |  |  |  |  |  |  |  |  |  |  |
| Score at A_1_ | |  | 0.01174 | 0.00020 |  |  |  |  |  |  |  |  |  |  |
| Retest (ΔA_2_-A_1_) | |  | 0.01188 | 0.00004 | 0.00082 |  |  |  |  |  |  |  |  |  |
| Age at A_1_ | |  | -0.09857 | -0.00013 | -0.00014 | 0.00116 |  |  |  |  |  |  |  |  |
| Inheritance | |  | -0.19550 | -0.00049 | -0.00097 | 0.00305 |  |  | 0.13709 |  |  |  |  |  |
| Age of parent at onset | |  | 0.00156 | 0.00003 | -0.00004 | -0.00029 |  |  | -0.00120 | 0.00039 |  |  |  |  |
| CAG | |  | -0.20942 | -0.00028 | -0.00012 | 0.00139 |  |  | 0.00103 | -0.00013 | 0.00358 |  |  |  |
| **FAS** | | 1.245 |  |  |  |  |  |  |  |  |  |  |  |  |
| Intercept | |  | 19.08516 |  |  |  |  |  |  |  |  |  |  |  |
| Score at A_1_ | |  | -0.25961 | 0.01232 |  |  |  |  |  |  |  |  |  |  |
| Age at A_1_ | |  | -0.08986 | -0.00046 |  | 0.00095 |  |  |  |  |  |  |  |  |
| CAG | |  | -0.18331 | -0.00107 |  | 0.00136 |  |  |  |  | 0.00339 |  |  |  |
| **IS** | | 6.870 |  |  |  |  |  |  |  |  |  |  |  |  |
| Intercept | |  | 16.94631 |  |  |  |  |  |  |  |  |  |  |  |
| Score at A_1_ | |  | -0.05219 | 0.00054 |  |  |  |  |  |  |  |  |  |  |
| Age at A_1_ | |  | -0.07895 | -0.00003 |  | 0.00074 |  |  |  |  |  |  |  |  |
| Number of CAG repeats | |  | -0.19390 | 0.00009 |  | 0.00109 |  |  |  |  | 0.00307 |  |  |  |
| **TFC** | | 1.023 |  |  |  |  |  |  |  |  |  |  |  |  |
| Intercept | |  | 4.52855 |  |  |  |  |  |  |  |  |  |  |  |
| Score at A_1_ | |  | -0.34639 | 0.03321 |  |  |  |  |  |  |  |  |  |  |
| Retest (ΔA_2_-A_1_) | |  | -0.27989 | 0.03539 | 0.15364 |  |  |  |  |  |  |  |  |  |
| Age at A_1_ | |  | -0.00975 | -0.00085 | -0.00287 | 0.00043 |  |  |  |  |  |  |  |  |
| Inheritance | |  | -0.17175 | 0.00392 | 0.01383 | 0.00131 |  |  | 0.11296 |  |  |  |  |  |
