# Supplementary material for: How to Capitalize on the Retest Effect in Future Trials on Huntington’s Disease
Source: PLoS One. 2015 Dec 29;10(12):e0145842. doi: 10.1371/journal.pone.0145842 (PMC4703129; doi:10.1371/journal.pone.0145842)
Supplement: S1 Text — (DOCX) [file pone.0145842.s003.docx]

**S1 Text. Statistical explanation for the calculation of the 95% prediction interval (95%PI) for performance at A_3_, for each task**

Let *P* be the predicted performance of the patient at A_3_. The 95%PI is given by the following formula:

$$P \pm t_{1-\frac{\alpha}{2},df} \times\sqrt{\hat{\sigma}^{2} \left( 1+ \sum_{k} \sum_{l} X_{k}X_{l}M_{k,l} \right)}$$

Where $t_{1-\frac{\alpha}{2},df}$ is the student quantile of order $1-\frac{\alpha}{2}=0.975$ and *df* is the number of degrees of freedom, defined by *df=n-p-1,* where *n* and *p* are the number of subjects and variables in the model, respectively. It can be approximated by $t_{1-\frac{\alpha}{2},df}=2$; $\hat{\sigma}$ is the residual variance of the predicted model; *k* and *l* are the predictive factors for each task, from the following list: *intercept*, *score at A_1_*, *retest (ΔA_2_-A_1_)*, *age at A_1_*, *sex*, *education level*, *inheritance*, *age of parent at onset of disease*, *number of* *CAG repeats*, *time since onset* and *first symptom*; *M* is a matrix defined for each task in Supplementary Table 2. *X* is an observed characteristic of a future patient such that:

- *X_intercept_*=1;
- If the variable *k* is quantitative, *X_k_*=value of variable;
- *X_sex_* =1 if a man, 0 if a woman;
- *X_inheritance_*=1 if paternal inheritance, 0 if maternal inheritance;
- *X_first symptom =_ (X_cognitive_ , X_psychiatric_ )*=(0,0) if the first symptom was motor, (1,0) if it was cognitive and (0,1) if it was psychiatric.
